# The Speech of Souls

**Published:** 1895-08-03

**Authors:** 


### THE SPEECH OF SOULS.

PERIODICALLY, in alternation with a fit of materialistic negation, a wave of credulity passes over the world. Exhibitions of hypnotism become a part of private and public entertainments—exhibitions most trivial, often fraudulent, which, after the novelty of them has passed, are turned into ridicule and condemned as pure folly, invented by rogues and paid for by fools. It is certain that the mysterious and impalpable are always liable to be used by the unscrupulous for their own ends, but this can hardly be taken to prove that nothing exists which the average man has not seen, touched, or tasted. The Society for Psychical Research has investigated cases of apparition and hallucination, and the very incompleteness of their narratives, so different from the spick and span accuracy of the ghost-story of the magazines, make them appear the more convincing. Mr. Frank Podmore,* in dealing with the "evidence for telepathy," has done so honestly, not suppressing records of failure, not trimming and touching up instances of success to make them more convincing. In almost all cases the names of agents and percipients is given in full, and the experiments are generally related in their own words. While, therefore, it may be permitted to doubt the platform displays of the professional mesmerist and his companion, it is only reasonable to consider fairly the statements of those whom we believe to be honest and conscientious.

It is, of course, right to allow for the unconscious self-deception of humanity. If agent and percipient in an experiment in thought-transference meet and discuss their results before recording them, we may be fairly sure that in many cases the symmetry of the record will be improved and its accuracy impaired. And there is also the risk that the environment in which the experiment takes place may half consciously suggest to the percipient the agent's act or thought. Thus when two ladies, the agent a student at the London School of Medicine for Women, resolved to try if the percipient could see the object on which her friend fixed her attention at a given hour, it seems very natural that the percipient should see "scalpels," if she knew where the agent would be, and how probably employed at the hour chosen. Reasoning, though unconscious, inference would account for this, as well as thought-transference. But, in a subsequent experiment, the percipient saw a pair of gloves, "certainly a size larger than worn by R. C. D. or myself," and at the same time the agent, Miss Despard, had picked up "a pair of rather old, tan-coloured gloves, purposely not taking a pair of her own." This may be regarded by some as a successful instance of thought-transference.

It is true that these arranged experiments are not so impressive as the old unverified instances of vision, but when attempted by honourable people and carried out with strict avoidance of collusion, they tend to show that such a thing is possible as the transference of an idea, however trivial, from one mind to another. This fact, if proved, would make it easier to believe that where deep sympathy or affection exists between two people a thought might be conveyed from one mind to the other without conscious intention on either part. A very curious instance of sympathetic emotion is recorded by Archdeacon Bruce. He had gone to open a new church at Ebbw Vale, and after the ceremony he was walking through the vale, when over a provision shop he saw "one of those huge, staring Bovril advertisements—the familiar large ox-head." "I had seen fifty of them before," he says, "but something fascinated me in connection with this particular one. I turned to it, and was moved to address it in these, my *ipsissima verba*, 'You ugly brute, don't stare at me like that; has some accident happened to my wife?'" This was at twenty minutes past three, and before half-past three, Mrs. Bruce and her daughter, who were out driving, were thrown from their carriage. Mrs. Bruce says in her record of the incident, "The first thought that flashed across me as the accident happened was, 'What will he say?' My ruling idea then was to get home before my husband, so as to save him alarm."

A somewhat similar but sadder instance of this telepathic knowledge is given by Mr. E. A. Goodall, the artist, who, when in Italy, "awoke, as it seemed, at the sound of my own voice, saying, 'I know I have lost my dearest little May.' Another voice, which I in no way recognised: 'No, not May, but your youngest boy.'" The next mail brought news of the child's death, though when he left home a few days before all his family had been in good health.

These cases of individual impression are, though difficult to dispute, less remarkable than what are called collective hallucinations, when, that is, the same sight is seen or the same voice heard by more than one person at the same time. In one case a lady heard the voice of a little girl who had recently died, and to whom she had been much attached, calling her by her pet name, "Miss Boo." She was conversing with the child's mother at the time, but was not speaking of her, and when after a moment she recollected that it was no living voice she heard, she was about to continue her sentence, when the mother interrupted her, saying, "'Miss Newbold, did you hear that?' 'Yes,' I replied," says Miss Newbold's narrative. "'What was it?' and she said, 'My little child, and she called "Miss Boo."'" Neither the mother nor Miss Newbold had ever heard voices of any kind before, and although there was nothing strange in either thinking of the child whom both had loved, and who had died only a short time before, it is curious that both should have heard the voice at the same moment.

Are we to set such things aside as mere delusion? In most instances, especially with the professional clairvoyant who seems able to see visions and hear voices at will, we are justified in being sceptical. But such cases as we have quoted are apart from these, and appear to demand a complete investigation in regard to both mental state and physical surroundings. To be for ever listening for voices, or striving to pierce into things unseen, is foolish, and unfits the overstrung mind for the exercises of daily life, but we should be careful not to deny the possibility that anything exists beyond the tangible and material.

* "Apparitions and Thought Transference: An Examination of the Evidence for Telepathy." By Frank Podmore, M.A. The Contemporary Science Series. (London: Walter Scott, Limited, 24, Warwick Lane, Paternoster Row. Price 3s. 6d.)

